# Perioperative complications after pacemaker implantation: higher complication rates with subclavian vein puncture than with cephalic vein cutdown

**DOI:** 10.1007/s10840-022-01135-x

**Published:** 2022-02-02

**Authors:** Fuad Hasan, Sotirios Nedios, Zana Karosiene, Marvin Scholten, Bernd Lemke, Sabrina Tulka, Stephanie Knippschild, Susanne Macher-Heidrich, Heinz Jürgen Adomeit, Markus Zarse, Harilaos Bogossian

**Affiliations:** 1 Department of Cardiology, Elektrophysiology, and Angiology, Klinikum Lüdenscheid, Paulmannshöherstr. 14, 58515 Lüdenscheid, Germany; 2 University Witten/Herdecke, Witten, Germany; 3 Department of Electrophysiology, Heart Center, University of Leipzig, Leipzig, Germany; 4 Faculty of Health, Institute for Medical Biometry and Epidemiology, Witten/Herdecke University, Witten, Germany; 5 Medical Chamber North Rhine, Düsseldorf, Germany; 6 Medical Chamber Westphalia, Münster, Germany; 7 Department of Cardiology and Rhythmology, Ev. Krankenhaus Hagen, Hagen, Germany

**Keywords:** Pacemaker implantation complication, Venous access, Cephalic vein cutdown, Subclavian puncture

## Abstract

**Purpose:**

The cephalic vein cutdown (CVC) and the subclavian puncture (SP) is the most common access for pacemaker implantation. The purpose of this study was to compare the peri-/postoperative complications of these approaches.

**Methods:**

A retrospective analysis of the quality assurance data of the state of North Rhine-Westphalia was performed to evaluate the peri-/postoperative complications of first pacemaker implantation according to the venous access. The primary endpoint was defined as the occurrence of one of the following: asystole, ventricular fibrillation, pneumothorax, hemothorax, pericardial effusion, pocket hematoma, lead dislocation, lead dysfunction, postoperative wound infection or other complication requiring intervention. Descriptive analysis was done via absolute, relative frequencies and Odds Ratio. Fisher’s exact test was used for comparison of the both study groups.

**Results:**

From 139,176 pacemaker implantations from 2010 to 2014, 15,483 cases were excluded due to other/double access. The median age was 78 years and the access used was CVC for 75,251 cases (60.8%) and SP for 48,442 cases (39.2%). The implanted devices were mainly dual-chamber pacemakers (73.9% in the CVC group and 78.4% in the SP group), followed by single-chamber pacemakers VVI (24.9% and 19.9% in the CVC and SP group respectively). There were significantly fewer peri/postoperative complications in the CVC group compared to the SP group (2.49% vs. 3.64%, p = 0.0001, OR 1.47; 95% CI 1.38–1.57).

**Conclusions:**

CVC as venous access for pacemaker implantation has significantly fewer peri/postoperative complications than SP and appears to be an advantageous technique.

## Introduction

The number of cardiac implantable electronic devices (CIEDs) has steadily increased over the past decade. Between 2007 and 2016, an increase in pacemaker implantation of 20% was reported in the ESC member countries. In 2016, a total of 547,586 PMs were implanted in 4022 centres across the ESC area [1].

There are several international guidelines on the indications for pacing therapy. However, recommendations on the different surgical techniques for pacemaker implantation were not available until recently [2], which is generally learnt by a proctoring process during training [3].

Since Fuhrmann reported on the successful temporary transvenous endocardial pacing via the brachial vein in 1959 [4], the technique for the transvenous endocardial insertion of the cardiac implantable electronic devices (CIEDs) has developed over different phases. As a result, cephalic vein cutdown (CVC) and subclavian puncture (SP) are currently widely used techniques for lead insertion [5, 6]. The choice of the venous access technique used depends on operator experience.

However, which of the two techniques is safer and preferred during CIED implantation is still being debated. In order to evaluate which of the two approaches is safer, we performed this retrospective analysis of a large patient database.

## Methods

### Study design

In the period between January 2010 and December 2014, 139,176 pacemakers were implanted in 143 centres in the state of North Rhine-Westphalia in patients who were at least 18 years old.

The data acquisition took place within the framework of the mandatory quality assurance according to SGB V (§135 to §137) at all clinics in Germany that implant pacemakers. These data were analysed retrospectively to compare the peri-/postoperative complications of pacemaker implantation depending on venous access (CVC vs SP). The study protocol was approved by the Ethics Committee of the University Witten/Herdecke.

**Fig. 1:**
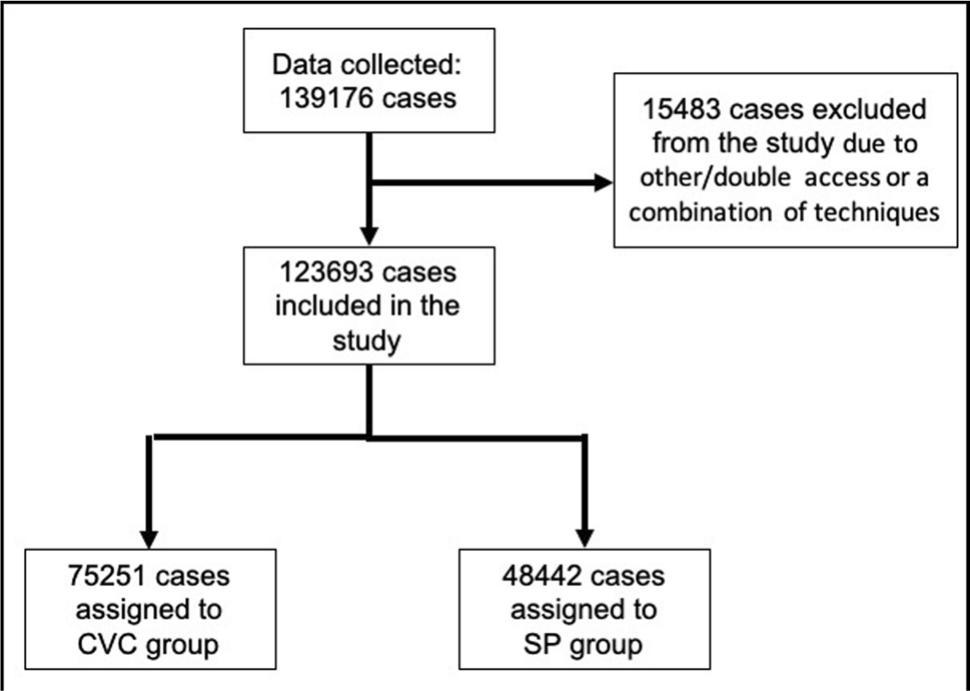
Study design

Cases (n = 15,483) with the use of both CVC and SP together, different access and/or a combination of several techniques were excluded from the study. Of the remaining 123,693 implantations, 75,251 cases were included in the CVC group and 48,442 cases in the SP group (Fig. 1).

The primary endpoint of this study was defined as the occurrence of one of the peri- or postoperative complication during pacemaker implantation.

The peri-/postoperative complication was defined in quality assurance as one of the following: asystole, ventricular fibrillation, pneumothorax, hemothorax, pericardial effusion, pocket hematoma, lead dislocation, lead dysfunction, postoperative wound infection, or other complication requiring intervention.

### Statistical analysis

As the study was as a retrospective analysis on a full data set, no sample size calculation was performed. As the primary endpoint was binary, descriptive analysis was done via absolute and relative frequencies and supported by the Odds Ratio. Fisher’s exact test was used for comparison of the both study groups. As the reported results are part of a greater study the level of significance was set to 2.5% (Bonferroni adjusted). Any further data analysis was performed using descriptive methods. Results of continuous variables were presented via minimum, quartiles, median, maximum and supported by boxplots. Analysis of categorial data was done by calculating absolute and relative frequencies supplemented by barplots. The statistical evaluation was performed using the statistical and analysis software R Core Team (2020). R: A language and environment for statistical computing. R Foundation for Statistical Computing, Vienna, Austria. URL: https://www.R-project.org.

## Results

The whole data set consisted of 139,176 patients. Of these 123,693 patients (88.8%) were included in the data analysis as they underwent either CVC or SP. The remaining patients were excluded due to the combination technique or the use of a different access.

In 75,251 (60.8%) patients, the CVC was used and in 48,442 (39.2%) patients the SP was used. Baseline characteristics of both study populations are summarized in Table 1. The mean age of the patients in both groups was 77 years. In both groups, the majority of patients had an ASA classification 2–3 and normal LV function or slight LV dysfunction (EF > 50%). Dual and single chamber pacemakers were the most commonly used devices (98%) in both groups (Table 1).

**Table 1:** Baseline characteristics

PM: (n = 123,693)	CVC	SP
PM 2010–2014	75,251(60.8%)	48,442 (39.2%)
Gender		
Male	40,339 (53.60%)	24,300 (50.16%)
Female	34,912 (46.39%)	24,142 (49.83%)
Age in years (mean)	77 ± 7	77 ± 7
ASA classification		
1 = healthy person	5222 (6.93%)	2885 (5.95%)
2 = mild systemic disease	33,391 (44.37%)	21,524 (44.43%)
3 = severe systemic disease	33,897 (45.04%)	22,491 (46.42%)
4 = severe systemic disease that is a constant threat to life	2637 (3.50%)	1495 (3.08%)
5 = moribund patient	104 (0.13%)	47 (0.09%)
Systolic left ventricle function (EF = ejection fraction)		
EF: not known	7199 (9.56%)	6332 (13.07%)
EF: > 50%	58,838 (78.18%)	36,632 (75.62%)
EF: 50–36%	8469 (11.25%)	4631 (9.55%)
EF: ≤ 35%)	745 (0.99%)	847 (1.74%)
PM system:		
1 = VVI	18,733 (24.89%)	9670 (19.96%)
2 = AAI	141 (0.18%)	51 (0.10%)
3 = DDD	55,628 (73.92%)	37,986 (78.41%)
4 = VDD	507 (0.67%)	106 (0.21%)
5 = CRT-System with atrial lead	185 (0.24%)	535 (1.10%)
6 = CRT-System without atrial lead	45 (0.05%)	77 (0.15%)
9 = other	12 (0.01%)	17 (0.03%)

Although the proportion of implanted CRT systems in the SP group was three times more common than in the CVC group, this difference in the two groups does not affect the overall result because all implanted CRT systems are only 0.68% of all the implanted pacemakers included in the study.

The CVC group had a complication rate of 2.49% (n = 1879). The complication rate in the SP group was 3.64% (n = 1765). The difference of 1.15% had statistical significance with a p-value < 0.0001. The Odds Ratio of SP in comparison to CVC was 1.47 (95% CI: 1.38–1.57, p < 0.001).

**Fig. 2:**
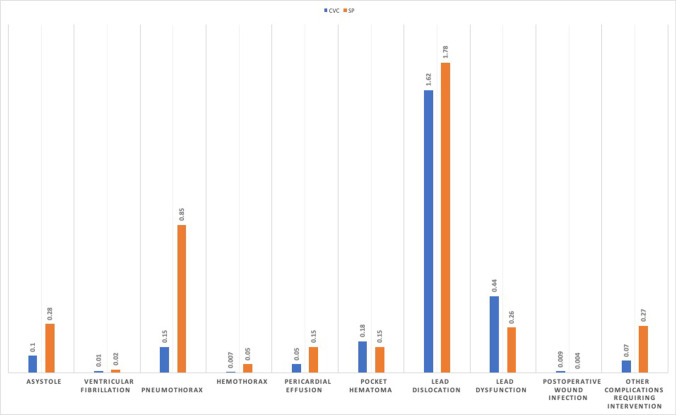
Peri-/postoperative complications

All peri-/postoperative complications are summarized in Table 2. In both groups, lead dislocation (CVC group: 1.62%; SP group 1.78%) was the most common complication. Pneumothorax with 0.85% in the SP group comes in second place and is 5 times more common than in the CVC group (0.15%). Ventricular fibrillation, hemothorax, and postoperative wound infection (0.009% in the CVC group and 0.004% in the SP group) were among the rare complications (Fig. 2).

**Table 2:** Peri-/postoperative complications

	CVC	SP	P-Value	OR	95% CI
PMs 2010–2014	75,251(60.8%)	48,442 (39.2%)			
Peri-/postoperative complications:	**1879 (2.49%)**	**1765 (3.64%)**	** < 0.0001**	**1.47**	**1.38–1.57**
Asystole	78 (0.10%)	138 (0.28%)	< 0.0001	2.75	2.08–3.63
Ventricular fibrillation	15 (0.01%)	13 (0.02%)	0.4	1.34	0.64–2.82
Pneumothorax	117 (0.15%)	412 (0.85%)	< 0.0001	5.5	4.48–6.76
Hemothorax	6 (0.007%)	26 (0.05%)	< 0.0001	6.7	2.77–16.36
Pericardial effusion	38 (0.05%)	74 (0.15%)	< 0.0001	3.0	2.04–4.47
Pocket hematoma	140 (0.18%)	73 (0.15%)	0.14	0.8	0.61–1.07
Lead dislocation	1224 (1.62%)	866 (1.78%)	0.03	1.10	1.00–1.20
Lead dysfunction	333 (0.44%)	129 (0.26%)	< 0.0001	0.6	0.49–0.73
Postoperative wound infection	7 (0.009%)	2 (0.004%)	0.29	0.44	0.09–2.13
Other complications requiring intervention	58 (0.07%)	132 (0.27%)	< 0.0001	3.54	2.60–4.82

**Fig. 3:**
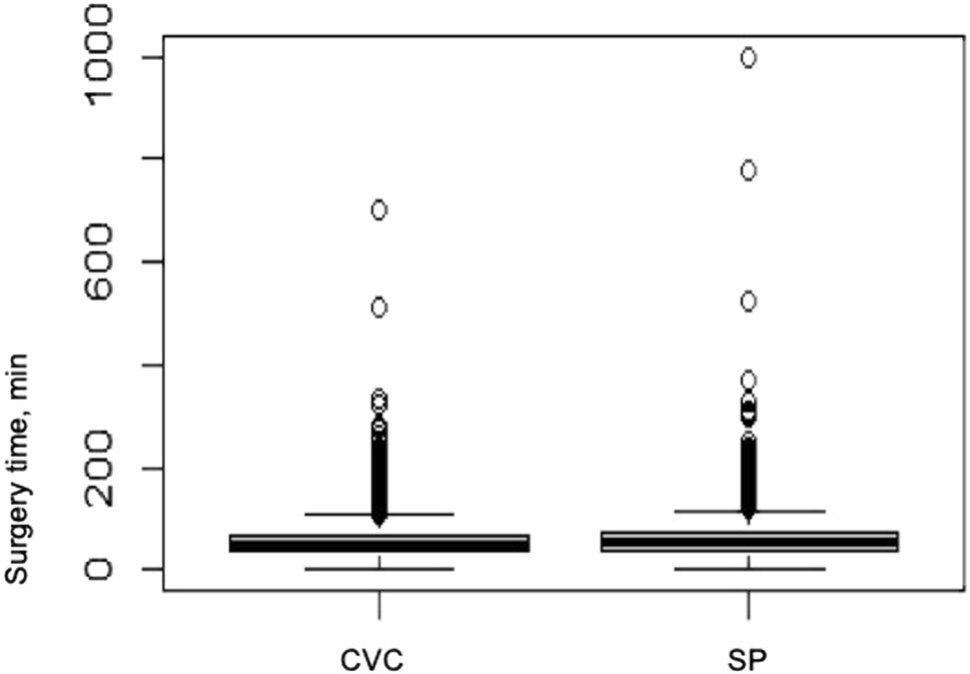
Surgery duration in min

**Fig. 4:**
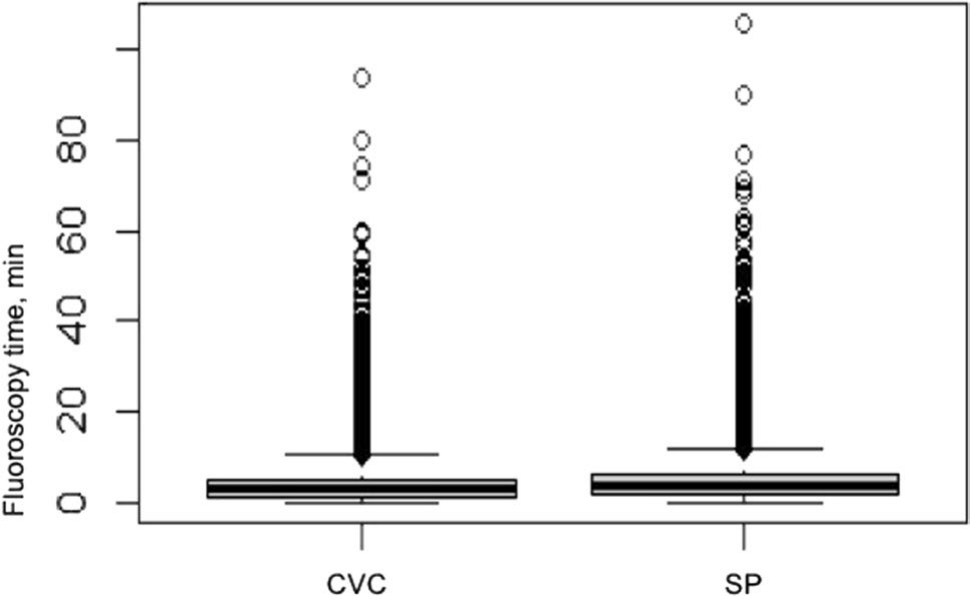
Fluoroscopy time in min

The duration of the surgery and the fluoroscopy time are summarized in Table 3. The pacemaker implantation using the SP technique took a median of 5 min longer than the implantation using the CVC technique. Figure 3 shows the duration of the surgery in both groups. The median time of fluoroscopy in the SP group was also 0.6 min longer than in the CVC group. Figure 4 shows the fluoroscopy time in both groups. Both the surgery time and the fluoroscopy time contain isolated values that appear unrealistic and because these are only individual cases, they have no influence on the overall result.

**Table 3:** Surgery duration and fluoroscopy time

	**CVC**	**SP**
Surgery duration, min (median)	49	54
Fluoroscopy time, min (median)	2.9	3.5

### Subgroup analysis

#### Number of pacemaker leads

To assess the influence of the number of pacemaker leads on the complication rate, the complication rate in both groups was determined depending on the number of pacemaker leads. Two or more pacemaker leads (DDD/CRT-subgroup) were implanted in the majority of procedures (74.2% in CVC group and 79.6% in SP group). In contrast, the proportion of procedures with a single lead was only 25.7% in the CVC group and 20.2% in the SP group.

In the single lead subgroup, 505 (2.6%) events occurred in 19,381 CVC procedures. These were significantly fewer than 333 (3.38%) events in 9827 SP procedures (p value = 0.0001). Likewise in the DDD/CRT subgroup with 1372 (2.45%) events in 55,858 CVC procedures, significantly fewer events were registered than in the SP group with 1427 (3.69%) events in 38,580 SP procedures (p value < 0.0001).

No statistically significant difference can be observed when comparing the two subgroups within the main group (p value 0.25 within the CVC group and p value 0.14 within the SP group). The procedures and the complication rate of both subgroups (single lead and DDD/CRT) are summarized in Table 4.

**Table 4:** Complication rate depending on the number of pacemaker leads

n = 123,693	CVC; n = 75,251	SP; n = 48,442	P-value	OR	95% CI
	Complications/Procedures	Complications/Procedures			
Single lead	505/19381 (2.6%)	333/9827 (3.38%)	0.0001	1.31	1.13–1.50
DDD and CRT	1372/55858 (2.45%)	1427/38580 (3.69%)	< 0.0001	1.52	1.41–1.64

#### Access-dependent centre experience

Except for 4 centres, all of the remaining 139 centres have used both methods for the transvenous endocardial insertion of the CIEDs. In the two centres where only the SP method was used, 3 (1.11%) events occurred in 268 procedures performed; the other two CVC centres performed 231 procedures with a total of two events (0.86%).

The centres (n = 143) were divided into three groups according to the most commonly used technique (group 1 for CVC and group 3 for SP) and the expertise of the centre. Group 1 included centres (n = 77, 54%) with at least 2/3 (66.6%) of the procedures performed with CVC rather than SP. Group 3 consists of centres (n = 20, 14%) with at least 2/3 (66.6%) of the procedures performed with SP. Group 2 consists of centres (n = 46, 32%) that have a balanced experience in both methods, so that both the CVC proportion and the SP proportion are between 33.3% and 66.6%. Table 5 shows the groups of the centres according to their expertise with the associated procedures and the complications that have occurred. While the statistically significant difference in the complication rate between the CVC and the SP group can still be observed in groups 1 and 2, this significance can no longer be demonstrated in group 3 with the predominant SP experience.

**Table 5:** Procedures and complication rates in different categories of implantation centres according to the most-commonly used venous-access/expertise of the centre (group 1 mostly using CVC, group 3 mostly using SP)

Group	Total	CVC	SP	P	OR	95% CI
Procedures, n (%)	123,693 (100)	75,251 (60.8)	48,442 (39.2)			
Group 1:	CVC ≥ 66.6% of the procedures					
Centres, n (%)	77 (53.8)	77 (100)	75 (97.4)			
Procedures, n (%)	57,660 (46.6)	43,938 (76.2)	13,722 (23.8)			
Complications, n (%)	1661 (2.88)	1156 (2.63)	505 (3.68)	< 0.0001	1.41	1.27–1.57
Group 2:	CVC/SP 33–66% of the procedures					
Centres, n (%)	46 (32.2)	46 (100)	46 (100)			
Procedures, n (%)	53,907 (43.6)	29,485 (54.7)	24,422 (45.3)			
Complications, n (%)	1601 (2.96)	675 (2.29)	926 (3.79)	< 0.0001	1.69	1.53–1.87
Group 3:	SP ≥ 66.6% of the procedures					
Centres, n (%)	20 (14)	18 (90)	20 100)			
Procedures, n (%)	12,126 (9.8)	1828 (15.1)	10,298 (84.9)			
Complications, n (%)	382 (3.15)	48 (2.62)	334 (3.24)	0.16	1.24	0.91–1.68

## Discussion

Transvenous pacing is one of the most important innovations in cardiac pacing [7, 8]. It followed the development of an introducer, a peel-away sheath, described by Littleford et al. that encouraged routine pacemaker implantation without direct approach to any vein and contributed to the fact that non-surgeons increasingly learned and performed pacemaker implants [9].

Further improvements and developments in surgical techniques, pacemaker devices and pacemaker leads [7, 10], as well as an ageing population with the associated expansion of indication spectrum, explain the continuous increase in the annual pacemaker implantations and the increase in the number of implanting centres.

Despite the impressive developments in pacemaker therapy, complications associated with pacemaker implantation are still observed [11–14]. Analysing big data from quality assurance data or pacemaker register should help in finding out the approach with the lowest complication rate.

Our data in a large, unselected population shows an overall complication rate of 2.9% (3644 events in 123,693 procedures). The rate of all complications was very low in our study compared with previous reports [11–14]. The reason for this is that only those complications that required specific interventions were reported. Complications, such as pneumothorax, pericardial effusion, or haematoma that were managed conservatively were not reported. Furthermore, our study only represents the short-term complications in the first implantation of pacemakers.

When comparing the complication depending on venous access, our study with 1879 events of 75,251 implantations (2.49%) in the CVC group shows a significantly lower complication rate in the CVC group compared with 1765 events of 48,442 implantations (3.64%) in the SP group. The superiority of the CVC with respect to the complication as pneumothorax and lead failure compared to SP has also been demonstrated in two recently published meta-analyses [15, 16]. Our study with the large data differs in that not only individual complications were considered, but all peri-/postoperative complications that require intervention.

We determined statistical significance for the total complication rate of both groups. The events in the individual complications such as postoperative wound infection (only 2 in the CVC group) are so small that the statistical significance cannot be proved absolutely certain. The most common complication in both groups is lead dislocation, followed by pneumothorax. Both complications occurred more in the SP group, which is in agreement with previous studies [15, 16]. This tendency is also found in the complications, asystole, ventricular fibrillation, hemothorax, pericardial effusion, and other complication requiring intervention. In contrast, there was a higher percentage of lead dysfunction, pocket hematoma, and postoperative wound infection in the CVC group compared to the SP group.

The subgroup analysis of the centres according to their venous access expertise shows that over 50% of the centres had predominant experience with CVC, while the centres with predominant SP experience were presented with only 14%. The statistically significant difference in the complication rate between the CVC and the SP group could no longer be demonstrated in the subgroup with predominantly SP experience, so that the dominant experience could possibly have an influence on the overall result.

In a prospective randomised study, Calkins et al. demonstrated that the placement of endocardial pacemaker and defibrillator leads using the extrathoracic subclavian vein guided by contrast venography had a higher initial success rate compared to CVC with no difference in the incidence of complications [17]. The information in our retrospective studies does not contain any information on whether the subclavian puncture was performed extrathoracically or intrathoracically. In addition, the years of the study (2010–2014) encompass a period before the routine use of venographic methods for axillary vein access and ultrasound-guided venous access. As such the differences between the cutdown and Seldinger methods may be less in the current era.

Recently, the puncture of the axillary vein for lead insertion of cardiac implantable electronic devices has recently become more important, after several studies have shown the superiority of the axillary access in terms of complications compared to the SP and comparable to the CVC with a high success rate of the axillary access [15, 18–20].

## Limitations

The data collection of the quality assurance programme only included inpatient pacemaker implantations; outpatient pacemaker implantations were not considered. Because the data collection ends with the discharge of the patient from the hospital, the documented complications only represent the short-term complications. The already collected data do not provide us with any information on whether the operator’s expertise, the requirements in their own centre or the anatomical and clinical characteristics of the patient played a role in the choice of venous access. A further potential bias is that, our study has no randomization; therefore we have an imbalance in single observational characteristics (e.g. those undergoing CVC were more likely to have single-lead devices and preserved LVEF). Since our data encompass a period before the routine use of venographic methods for axillary vein access and ultrasound-guided venous access, the differences between CVC and improved puncture techniques may be less in the current era.

## Conclusion

Our retrospective data analysis demonstrated that the cephalic vein cutdown (CVC) as a venous access for pacemaker implantation has significantly fewer peri-/postoperative complications compared with the subclavian puncture (SP). Therefore, the CVC should be learned by all pacemaker implanters and appears to be an advantageous technique for the transvenous endocardial insertion of the cardiac implantable electronic devices.
